# The Role of Polarizability in Isoelectronic Ions: The Case of Pseudohalides

**DOI:** 10.3390/molecules30020323

**Published:** 2025-01-15

**Authors:** Mert Acar, Duccio Tatini, Barry W. Ninham, Pierandrea Lo Nostro

**Affiliations:** 1 Department of Chemistry “Ugo Schiff” and CSGI, University of Florence, 50019 Sesto Fiorentino, Italy; mert.acar@unifi.it; 2 Department of Biotechnologies, Chemistry and Pharmacy, University of Siena, 53100 Siena, Italy; duccio.tatini@unisi.it; 3 Materials Physics (Formerly Department of Applied Mathematics), Research School of Physics, Australian National University, Canberra, ACT 2600, Australia

**Keywords:** Hofmeister series, pseudohalides, polyatomic ions, polarizability, hydration, ion shape

## Abstract

Specific ion effects are widespread and have been studied for over a century, yet they remain poorly understood. Terms like “kosmotropes” and “chaotropes” are convenient rules of thumb but the frequent reversal of the Hofmeister series implies their limitations. Polarizability is often used to classify ions, with kosmotropes considered low in polarizability and chaotropes high. However, for polyatomic ions, this framework becomes misleading. The anisotropic nature of polarizability in polyatomic ions plays a decisive role in shaping their behavior. In this work, we study pseudohalides (KOCN, KSCN, and KSeCN) aqueous solutions to explore these effects. We evaluate properties of these anions through experimental measurements of conductivity, density, viscosity, infrared spectra, and polarizability. Our results demonstrate that, even for linear isoelectronic polyatomic ions, the anisotropy of polarizability governs their hydration behavior.

## 1. Introduction

Hofmeister effects are a complex and intriguing phenomenon in which the distinct nature of ions profoundly influences the physicochemical properties of their solutions and dispersions, e.g., solubility, viscosity, surface tension, density, conductivity, heat capacity, activity coefficient, osmotic pressure, and many more [1,2,3]. While at dilute concentrations electrostatic forces dominate, at moderate to high concentrations, e.g., for *c* > 0.15 M, specific ion effects emerge [2,4]. These effects are widespread and play important roles, especially in biology and chemistry. Although the first effect of salts was observed by John Dalton in 1840 while studying the volume changes of aqueous salt solutions, Franz Hofmeister systematically studied their effects at the end of the XIX century in his works on protein precipitation. After ranking the effects of several different salts on the stability of hen egg yolk albumin dispersions in water, the “Hofmeister series” was first established [1,2,5]. Since then, the emergence of specific ion effects was recognized in a wide array of systems, including bulk solutions, soft matter systems, interfacial phenomena, polar organic solvents, oscillating reactions, enzymatic activity, and many others [1,2,3,6,7,8,9,10,11,12].

Despite a plethora of investigations and literature reports carried out for more than a century of research, the mechanisms underlying these effects remain only partially understood. It is clear that classical models such as those derived by Debye and Hückel [13], Born [14,15], and Derjaguin–Landau–Verwey–Overbeek (DLVO) [16] fail to explain the experimental results and to predict the behavior of different salts in solution, especially at moderate or high concentrations [1,2,17]. While the inclusion of many-body interactions, quantum dispersion forces, and solute-specific hydration effects improved our understanding, a comprehensive theory is still lacking [2].

In the framework of specific ion effects, ions are usually classified either as *kosmotropes* or *chaotropes* [18]. These terms derive from the very first studies, conducted by J. L. M. Poiseuille, on the viscosity of aqueous salt solutions and published in 1849 [19]. It was observed that kosmotropes make their solutions more viscous than plain water at the same temperature, while chaotropes make them more fluid. Nowadays we argue that kosmotropes stabilize hydrogen-bonding networks in water and promote a more ordered local structure. These ions typically have high charge density and strong hydration; therefore, they reduce the entropy of water and stabilize macromolecular structures by favoring hydrophobic interactions, e.g., the native conformation of proteins. Common kosmotropes include ions such as SO_4_^2−^, F^−^, Li^+^, and Mg^2+^. In contrast, chaotropes are supposed to disrupt the hydrogen-bonding network in water and increase its entropy by weakening intermolecular interactions. They destabilize the folding of macromolecules by interfering with hydrophobic interactions and solubilizing nonpolar regions. Some examples include I^−^, SCN^−^, and NO_3_^−^.

The use of this terminology should be handled with care as ion effects vary depending on the specific system and conditions [20,21,22,23]. In fact, anomalies in the Hofmeister series or its reversals are frequently reported [6,24,25,26].

An important case where these terms remain somehow blurry is polyatomic ions. Unlike monoatomic spherical ions (such as halides), polyatomic ions often have anisotropic charge distributions, and therefore their polarizability is not uniform but depends on the direction in which it is calculated. As a matter of fact, the molecular structure and shape of polyatomic ions are of utmost importance in determining their interaction with the solvent and other substrates. For example, ions like nitrate and acetate may behave differently at surfaces or interfaces depending on their direction of approach. In a previous study we confirmed that isoelectronic halates (ClO_3_^−^, BrO_3_^−^, IO_3_^−^) have reversed trends in their kosmotropicity with respect to their corresponding halides (Cl^−^, Br^−^, I^−^) [27]. In fact, while iodate exhibits a strong kosmotropic character despite being the most polarizable ion in the series, chlorate acts as a chaotrope [27]. This reversal compared to halides underlines that although the three anions are isoelectronic, that is, they bear the same number of electrons in the outer shell, the different charge distribution results in different hydration properties [27,28,29].

In this study, we investigate another series of polyatomic isoelectronic ions (with 16 outer electrons), chalcogenocyanates that are archetypical pseudohalides, i.e., cyanate (OCN^−^), thiocyanate (SCN^−^), and selenocyanate (SeCN^−^). These ions mimic the chemical behavior of halides and share similarities as they form salts, acids, and complexes and often act as halogen substitutes in chemical reactions [30,31,32]. However, while halides are spherical and monoatomic, and halates have a trigonal bipyramidal geometry and contain double X=O bonds, pseudohalide anions considered in this work (as potassium salts) possess a linear geometry where either O, S, or Se are bound to the CN group.

**Scheme 1:**
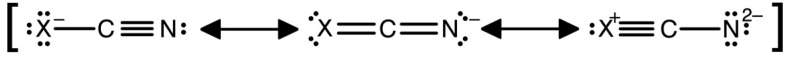
Resonance structure of pseudohalides XCN^−^, with X = O, S, or Se [30].

Cyanate’s ground-state configuration is 1σ^2^, 2σ^2^, 3σ^2^, 4σ^2^, 5σ^2^, 6σ^2^, 1π^4^ [30]. In contrast, the ground states of thiocyanate and selenocyanate exhibit higher complexity due to their core electrons and empty *d* orbitals [30]. Their structure is stabilized by resonance (see Scheme 1) [30].

Table 1 shows the partial charges on the ions derived from literature data [33] and calculations using MolCalc [34]. The chalcogen atom partial charges are consistent across different sources. Thiocyanate ion has the highest partial negative charge. As the chalcogen atom changes from oxygen to selenium, the carbon atoms exhibit an increasing negative charge, while the nitrogen atoms show a decreasing negative charge. The reported values for the charges on the carbon and nitrogen atoms of the cyanate differ, but the general trend is consistent. Furthermore, no published data were found for the SeCN^−^.

The variations in charge distribution among the chalcogenocyanates, which depend on the electronic structure of each constituent chalcogen atom, result in anisotropy in their polarizability.

Our question is how does the polarizability of isoelectronic polyatomic ions influence their ion-specific behavior, and to what extent does it serve as a determining factor?

Here we present several experimental data on the conductivity, density, viscosity, refractive index, and infrared spectroscopy features of these salts in water solutions and discuss their behavior in terms of polarizability. It is important to recall that the contribution brought by the potassium counterions will be assumed to be the same, independent from the anion.

## 2. Results

The results will be presented in different sections in the following order: conductivity, density, viscosity, infrared spectroscopy, and refractive index. The partial molar volumes and the polarizabilities will be calculated from the density and the refractive index values, respectively.

### 2.1. Conductivity

**Figure 1:**
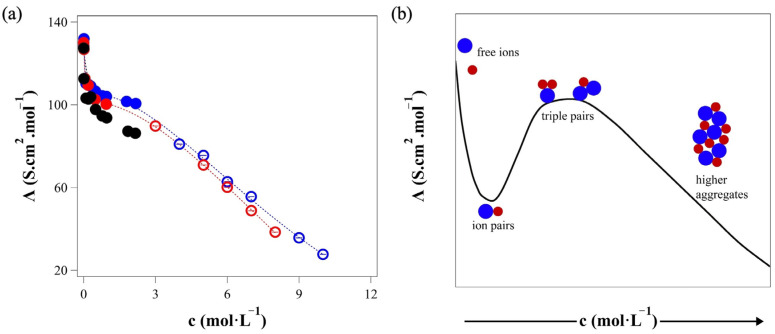
(**a**) Molar conductivity (*Λ*) vs. concentration (*c*, in molar units) plots of potassium cyanate (●), thiocyanate (●) and selenocyanate (●) solutions. Open circles are values taken from ref [35]. Dotted lines are guides for the eye. The experimental error on the molar conductivity values is ±0.1; (**b**) a sketch of molar conductivity trends vs. concentration showing the formation of ion aggregates as the salt concentration increases. In this plot the concentration scale is only illustrative and the specific behavior of a salt changes depending on its composition and on the pairing/association features of its ions. Adapted with permission from ref. [36], Copyright 2019, Elsevier.

The conductivity (*κ*, in mS·cm^−1^) and molar conductivity (*Λ*, in S·cm^2^·mol^−1^) for each salt solution at 25 °C are reported in Table S1 (see Supplementary Material). The plots of *Λ* vs. *c* are shown in Figure 1 and *κ* vs. *c* in Figure S1.

The conductivity values reflect the different solvation, dissociation, and ion-pairing features of the three investigated salts. The variation of *Λ* as a function of concentration *c* reveals distinct ion transport behaviors.

The molar conductivity of KOCN solutions drops very sharply at low concentrations as expected for strong electrolytes that fully dissociate in water. At about 1 M, the ion-pair formation becomes detectable. In contrast, the solutions of KSCN and KSeCN show a more gradual decline in conductivity as concentration increases. The observed trend suggests incomplete dissociation of the two latter salts, likely due to the ion-pair formation or aggregation (see Figure 1b). The double and triple ion pair formations are not markedly visible, and the shape of the *Λ/c* curve suggests the formation of higher aggregates. The trends for *c* > 3 M are in line with the formation of higher aggregates. In conclusion, the conductivity study seems to indicate that KSCN and KSeCN possess a very similar behavior, different from that of KOCN.

Furthermore, using Equation (1) (see Section 4.2), the limiting molar conductivity values *Λ*^∞^ (in S·cm^2^·mol^−1^) of the salts were calculated and compared to similar values extracted from previous works (see Table 2) [37]. These values are the conductivities at infinite dilution and therefore are indicative of solute–solvent interactions only.

Our values align very well with those found in literature [37]. KSCN has the highest limiting molar conductivity, while KSeCN sits in the middle of the trend. The change in *Λ*^∞^ is nearly negligible between the three different salts, indicating that a similar dissociation mechanism takes place at extreme dilution.

### 2.2. Density

**Figure 2:**
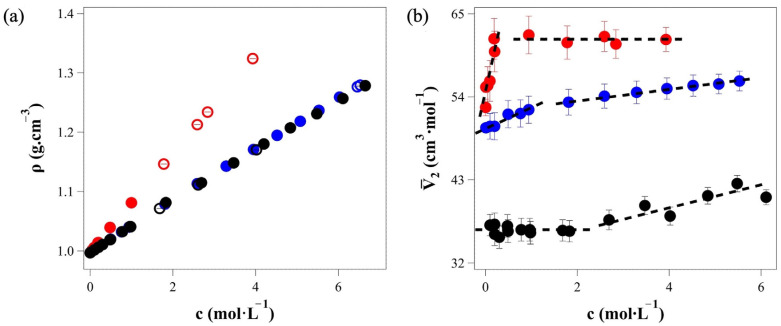
(**a**) Density (*ρ*, in g·cm^−3^) vs. salt concentration (*c*, in molar units) for potassium cyanate (●), thiocyanate (●), and selenocyanate (●) aqueous solutions at 25 °C. Blue open circles are values taken from ref. [38], black and red open circles from ref. [39]; (**b**) partial molar volumes ($\bar{V_{2}}$, in cm^3^∙mol^−1^) at various concentrations (*c*, in molar units). The dotted black lines indicate two different trends depending on the salt concentration regime.

Densities of aqueous salt solutions of KOCN, KSCN, and KSeCN were measured as a function of the concentration at 25 °C. The obtained values were used to calculate the apparent molar volumes (V2ϕ, in cm^3^∙mol^−1^), standard partial molar volumes (${\bar{V}}_{2}^{o}$, in cm^3^∙mol^−1^), and partial molar volumes ($\bar{V_{2}}$, in cm^3^∙mol^−1^) following the procedures described in the Materials and Methods section (see Equations (2)–(4)). All values are listed in Tables S2 and S3 in the Supplementary Material. The plots of density and partial molar volume are shown in Figure 2, while the plot for the apparent molar volumes vs. concentration is shown in Figure S2 in the Supplementary Material.

The densities of potassium cyanate and thiocyanate solutions show a similar linear trend with increasing salt concentration (see Figure 2a). On the other hand, the trend of *ρ* for potassium selenocyanate is different, with higher values at constant concentration. For KSeCN the slope of the *ρ*/*c* curve is much higher, and the trend begins to deviate from linearity around 2 M. Overall, the densities of three water salt solutions follow the sequence KSeCN > KSCN~KOCN.

Figure 2b shows the partial molar volume $\bar{V_{2}}$ of each salt solution as a function of the concentration.

The plots show two different trends for each salt and can be split between a low/moderate and a high concentration regime (see Figure 2b). In more detail, at concentrations below 1.83 M, KOCN has a relatively constant $\bar{V_{2}}$ value of 36 ± 2 cm^3^·mol^−1^, whereas at higher concentrations, a linear increasing trend appears. KSCN shows a similar behavior but starting from 0.98 M. For KSeCN the behavior is reversed with respect to KOCN: up to 0.19 M a quite steep linear increasing trend is observed, and at larger concentrations the partial molar volume reaches the value of 60 ± 2 cm^3^·mol^−1^ and remains constant at least up to 4 M.

These values can be explained by considering that the partial molar volumes reflect both solute–solvent and solute–solute interactions. For KOCN, the solute–solvent interactions dominate as shown by the constant $\bar{V_{2}}$ value up to very high concentrations (around 2 M). On the other hand, for KSCN and KSeCN, the discontinuity between the low and high concentration regimes occurs at much lower concentrations, about 1 M for KSCN and 0.2 M for KSeCN, respectively. The initial increasing linear trends found for KSCN and KSeCN are supposed to reflect solute–solute interactions. This is in line with the results obtained from the molar conductivity measurements, as they indicate the formation of ion pairs and larger ion aggregates. We recall here the law of matching water affinities (LMWA), according to which ions with similar solvation features (i.e., in kosmo–kosmo and chao–chao pairs) form ion pairs more readily [40,41]. In fact, K^+^ and SCN^−^ have similar Gibbs free energy change of hydration ∆*_hydr_G* (−295 and −280 kJ·mol^−1^, respectively) [42]. This means that thiocyanate possesses a high tendency to form ion pairs with potassium. The Gibbs free energy change of hydration for cyanate is −365 kJ·mol^−1^ [42]. The value of ∆*_hydr_G* for SeCN^−^ is not available in the literature and was estimated through MolCalc (−292 kJ·mol^−1^) [34]. In conclusion, we expect that the linear increment in the $\bar{V_{2}}$ values at low salt concentrations suggests the onset of ion pairing. KSeCN seems to favor to a higher extent the formation of ion pairs and aggregates with respect to KSCN based on the higher slope for the former.

This interpretation also explains the second part of the plot at higher concentrations. Ions that begin to exhibit solute–solute interactions at low concentrations and form already ion pairs and larger aggregates at low concentrations tend to stabilize the aggregates as the concentration increases further, with an increment in the number of aggregates. In contrast, KOCN, which does not readily form ion pairs, displays a steep slope at high concentrations. The observed behavior of ion pair formation can be summarized as KSeCN > KSCN > KOCN.

The standard partial molar volumes (${\bar{V}}_{2}^{o}$) are listed in Table 3 and compared to those found in the literature. Values obtained for KOCN and KSCN are in good agreement with previously published data, whereas a slightly lower value was found for KSeCN.

The data show a significant increase in ${\bar{V}}_{2}^{o}$ from KOCN to KSCN. KSCN and KSeCN possess comparable values, suggesting a similar behavior for the two salts. We recall that standard partial molar volumes reflect the solute–solvent interactions only. A low value for KOCN suggests that the solute contributes minimally to the solution’s volume presumably because of strong solute–solvent interactions. This hypothesis is further supported by its $\bar{V_{2}}$ values that remain constant even at 2 M.

The case of KSeCN is particularly interesting. In fact, O, S, and Se belong to the 16th group of the Periodic Table, sharing the same external electron configuration ns^2^np^4^. Moving down across the group, one would expect Se to contribute significantly to ${\bar{V}}_{2}^{o}$. The results suggest that despite its structure and size, other factors are probably at play that stabilize its interactions with the solvent.

### 2.3. Viscosity

**Figure 3:**
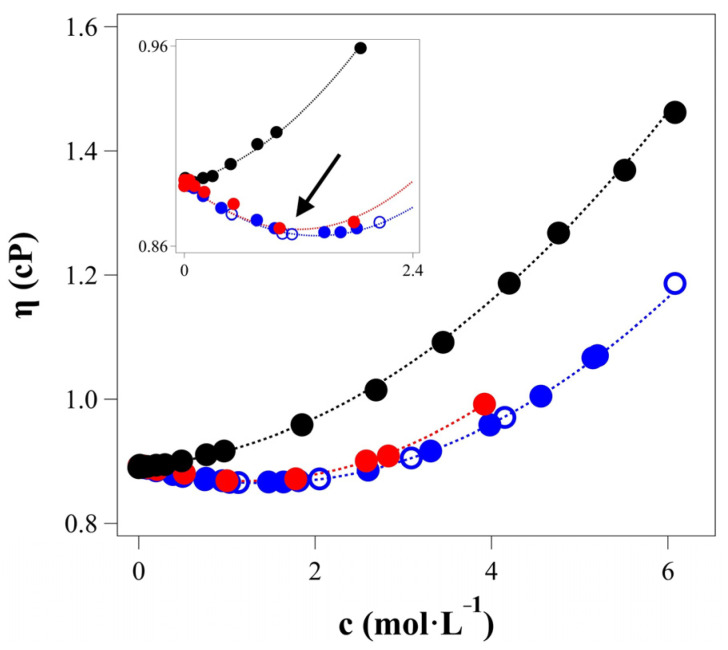
Viscosity (*η*, in mPa·s) as a function of the concentration (c, in mol·L^−1^) of potassium cyanate (●), thiocyanate (●), and selenocyanate (●) solutions at 25 °C. Open circles are the values taken from ref [38]. Dotted lines are the fitting curves from the extended Jones–Dole equation (see Equation (5)). Inset: the magnified plot of lower concentration region. The arrow indicates the viscosity break point for KSCN and KSeCN.

The viscosity (*η*, in mPa·s) of each salt solution as a function of the concentration at 25 °C is reported in Table S4 (see the Supplementary Material). Figure 3 shows the plots of these values fitted according to the extended Jones–Dole equation (see Equation (5)).

For KOCN the viscosity increases with increasing concentration. For KSCN and KSeCN, the viscosity plot can be divided into two parts with a minimum around 1 M. Chaotropic salts are known to decrease the viscosity of their solutions. However, this behavior is concentration dependent. As shown in the inset of Figure 3, in KSCN and KSeCN solutions the viscosity first decreases but then reverses its trend at higher concentrations. This effect is due to the crowding of the environment at higher concentrations and shows the onset of significant solute–solute interactions.

Some physicochemical properties (*f*) of solutions can be fitted by a semi-empirical equation $f=f_{0}\left(1+A\sqrt{c}+Bc\right)$, where *f*_0_ is the same property for plain water at the same temperature and *c* is the salt concentration. This occurs in the case of viscosity, apparent molar volume, optical rotation, enzymatic activity, and in other cases [6,43,44,45].

In the case of viscosity (see Equation (5)), *A* is the Falkenhagen coefficient and reflects electrostatic interactions. In fact, the $A\sqrt{c}$ term dominates at very low concentrations. *A* is also the only coefficient that can be calculated by theoretical means [46,47]. The second term is needed at higher concentrations, up to approximately 0.1 M. The *B* coefficient reflects the solute–solvent interactions and is ion specific. Its value is positive for kosmotropes and negative for chaotropes. For higher concentrations, more terms like *Dc*^2^ need to be added. The *D* coefficient is expected to depend on the solute–solute interactions, but no clear indication of its physical meaning is available at the moment [48].

Table 4 lists the extracted fitting parameters. KOCN has a *B* coefficient close to zero. Instead, KSCN and KSeCN have negative values, which is typical for chaotropic ions. Again, we find that the behavior of SeCN^−^ is similar to that of SCN^−^; in fact, they possess a pretty similar chaotropic nature.

The *D* coefficients are similar for all salts. However, based on the previous results, we cannot solely attribute this coefficient to solute–solute interactions, as KSCN and KSeCN show a tendency for ions pairing, but KOCN does not. The results found for the *D* coefficient seem to suggest that more complex interactions than just solute–solute ones, for example, those in large ionic aggregates, may be at play.

### 2.4. Fourier Transformed Infrared Spectroscopy (FTIR)

FTIR spectroscopy offers insights into the microscopic behavior of aqueous electrolyte solutions [49]. OH bending, CN stretching, and the deconvolution of OH stretching spectra of KOCN, KSCN, and KSeCN at 0.5, 1, 1.5, 2, and 2.5 molal concentrations and of water at 25 °C are shown in Figures S3–S9 in the Supplementary Material.

The OH bending bands at around 1650 cm^−1^ show insignificant changes. The absorption intensities arise with the increasing concentration of each salt. In the case of KOCN, no shifts are detected. For KSCN and KSeCN, there is a small red shift to lower frequencies with increasing salt concentration.

The bands of CN bonds are different depending on the salt. KOCN has its CN stretching band at ~2155 cm^−1^, a higher frequency with respect to those of KSCN (2065 cm^−1^) and KSeCN (2075 cm^−1^). This is expected, as oxygen is more electronegative and thus increases the CN bonding strength, while the less electronegative sulfur and selenium withdraw less electron density. For all salt solutions we observed a red shift with the increasing concentration, with a stronger effect in the case of KOCN. This can be assigned to the higher capacity of oxygen to interact with the water molecules. As a result, the CN bond weakens.

The water OH stretching band (~3400 cm^−1^) is responsive to ion–water interactions and to hydrogen bonding [49,50,51]. It can be divided into two to four components by a deconvolution process, where each component corresponds to water molecules in distinct hydrogen-bonding (HB) environments [49,50,51]. Low-frequency components correspond to water molecules (bound water) with stronger and shorter hydrogen bonds, while high-frequency components (free water) correspond to weaker and longer ones [49,50,51]. The deconvoluted OH stretching bands were assigned to four types of different water based on the work of Masuda et al. as follows [51]:

- Type I (~3550 cm^−1^), the smallest cluster and the longest mean hydrogen bonding distance;
- type II (~3400 cm^−1^), small cluster and a long mean hydrogen bonding distance;
- type III (~3230 cm^−1^), larger cluster and short mean hydrogen bonding distance;
- type IV (~3080 cm^−1^), the largest cluster and the shortest mean hydrogen bonding distance.

Water clusters are dynamic aggregates of water molecules connected by hydrogen bonds, forming structures from small rings to large networks [52,53]. Their size and behavior are influenced by temperature, pressure, the presence of solutes, and impact water’s properties [52,53].

**Figure 4:**
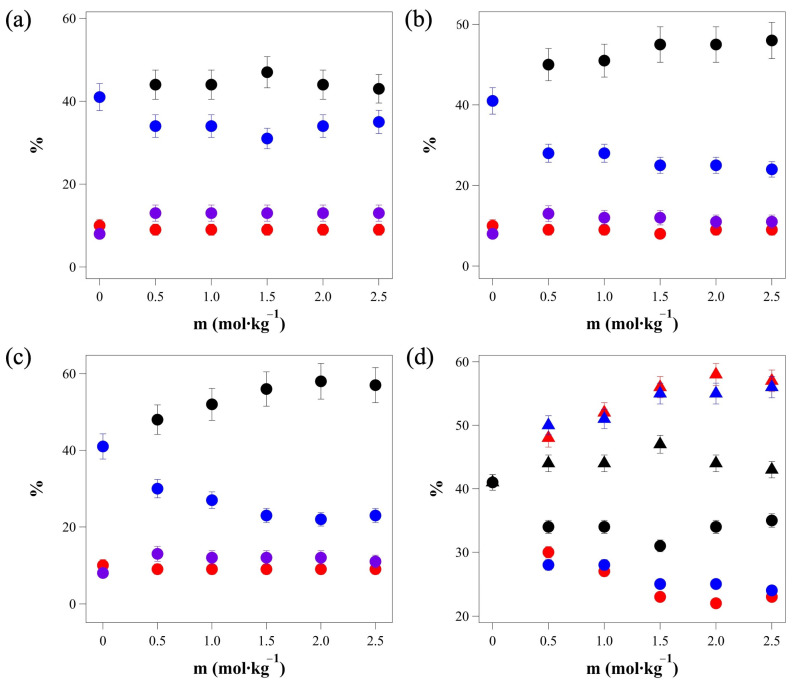
Fractions (%) of different types of water after the deconvolution of IR absorbance spectra of (**a**) KOCN; (**b**) KSCN; (**c**) KSeCN at various concentrations. Four different classes of water contributions, type I (━), type II (━), type III (━), and type IV (━) are shown; (**d**) shows the plots of comparison of Type II (triangles) and III (circles) between KOCN (black), KSCN (blue) and KSeCN (red).

Figure 4 shows the plots of the fraction of different types of water vs. the concentration of salts (in molal units). Type I and type IV water changes are negligible. This can be attributed to the fact that when water molecules are too far away from an ion, they do not sense the strong ionic electric field. The same applies when the water molecules are tightly bound to the ion; their dielectric constant is strongly lowered, and their response to the ionic electric field is rather weak.

The major changes are shown in the case of types II (small water clusters with weak hydrogen bonding) and III (large clusters with strong hydrogen bonding). Figure 4, panel d, shows that KSCN and KSeCN behave similarly. In fact, by increasing the concentration of the salts, the hydrogen bonding of the water molecules starts to convert from large clusters, with strong hydrogen bonding to small clusters with weak hydrogen bonding. This perturbation is in line with the chaotropic nature of the ions, as chaotropes are supposed to disrupt the structured hydrogen bond network of water, therefore reduce its cohesive properties, and increase the molecular disorder [2,18,54]. For concentrations above 1.5 m, the fraction of strong hydrogen bonding becomes approximately constant for KSCN and KSeCN. This occurrence may be due to the formation of higher aggregates that modify the overall hydration properties.

In the case of KOCN, the trends are very different. At first, the fraction of strong hydrogen bonds with large clusters decreases at 0.5 m concentration, becomes constant at 1.5 m, and then increases at higher concentrations. Overall, in the case of KOCN, the fractions of water molecules with different hydrogen bonding do not change significantly. The small variations are similar to those of KSCN and KSeCN. The small extent of the effect induced by KOCN may be justified by considering its low Jones–Dole *B* coefficient (0.009).

In conclusion, the FTIR experiments conducted on the KOCN, KSCN, and KSeCN solutions seem to suggest that the presence of the different ions induces detectable changes in the distribution of the different types of water molecules in solution, according to Masuda [51].

### 2.5. Refractive Index and Polarizability

**Figure 5:**
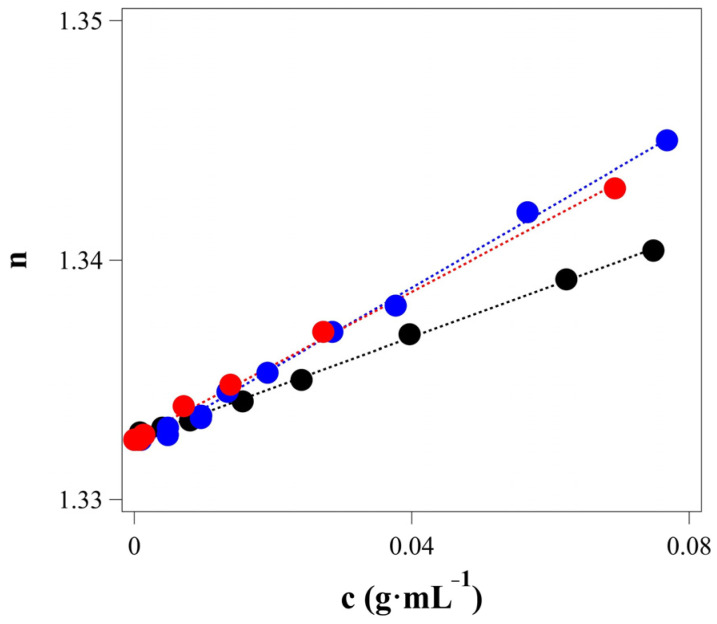
Refractive index (*n*) values at 25 °C as a function of the concentration (*c*, in g·mL^−1^) of potassium cyanate (●), thiocyanate (●), and selenocyanate (●) solutions. The fitting lines were obtained from Equation (6).

The refractive index values at 25 °C are listed in Table S5 (see the Supplementary Material). Figure 5 shows the refractive index vs. concentration for each salt solution. On the *x*-axis, the concentration is expressed in g·mL^−1^, a unit chosen based on the method used for calculating the ionic polarizabilities according to Equations (6) and (7).

When solutes are dissolved in a solvent, they alter the local electromagnetic field. The extent of this perturbation is based on various factors. The nature of the solute–solvent interactions is crucial, as they affect molecular packing, dipole moment, hydrogen bonding, and dispersion forces of the intervening species. Solutes that fit well into the solvent structure cause minimal disruption and lead to smaller changes in the refractive index. Also, weaker dipole interactions and low hydrogen bonding capacity result in little change in the refractive index with respect to the pure solvent.

In our study, the refractive indices of each salt up to moderate concentration regime (below 0.1 M) showed a linear increment. The increment was smaller in the case of KOCN, while it is greater and similar for KSCN and KSeCN.

The low slope of KOCN can be related to its rather low polarizability, weak hydrogen-bonding and dipole–dipole interactions, and therefore minimal perturbation of the solvent structure. This is in line with the very low Jones–Dole *B* coefficient (0.009) from viscosity measurements. In contrast, in the case of KSCN and KSeCN, the high slopes reflect higher polarizability and stronger solute–solvent interactions.

In Table 5 the refractive indices and polarizabilities of the salts are listed. The trend of the polarizabilities follows the sequence KOCN < KSCN < KSeCN, with a significant change in *α* between thiocyanate and selenocyanate. The implication of these results points toward a difference in hydration and polarization, which are usually thought to be related.

## 3. Discussion

The results obtained from different measurements indicate the presence of larger ionic aggregates in the case of KSCN and KSeCN in water, depending on the salt concentration. Usually, this kind of behavior is observed in polar aprotic solvents [55,56]. Instead, KOCN dissociates as a generic strong electrolyte and forms ion pairs only at high concentration.

The Debye screening length *λ*_D_ was calculated according to Equation (8). This equation is purely electrostatic and does not account for ion specificity, meaning that all 1:1 salts give the same value at a given concentration.

The Bjerrum length *λ*_B_ was calculated to be 7.2 Å using Equation (9). Lengths below this value are indicative of ion pairing propensity. In our case, the condition *λ*_D_ = *λ*_B_ is reached at around 0.2 M. This marks the concentration at which the immediate linear decrease in molar conductivity stops, indicating a good agreement with the theoretical end point of full ion dissociation.

FTIR spectroscopy shows that both closely bound water molecules and those farther away remain largely unaffected by the presence of ions. This finding is particularly significant as the literature reports an extensive debate on the range of influence of ions on the structure of the solvent [57,58,59,60,61,62,63,64,65,66,67,68]. While some theories propose that ions induce long-range ordering or disordering of the water network [57,58,59,60,61,62,63,64,65,66,67,68], our results suggest otherwise, in line with more recent results.

However, the water clusters at intermediate range (Types II and III) are significantly perturbed by the presence of ions. The trend of KSCN and KSeCN supports the role of ion pairing and higher aggregate formation. In fact, the increasing concentrations lead to an increase in weaker hydrogen bonding of water at the expense of the stronger HB cluster network. The presence of large aggregates with low electron density can indeed behave as a whole chaotrope and thus disrupt the HB network of water. The slight increase in stronger HB detected at the highest concentrations used in this study can be attributed to the exclusion of aggregate bodies, which then allow water to form more hydrogen bonds. A simulation study has shown that KSCN forms clusters in aqueous solutions [69]. In contrast, KOCN causes only minor changes in the HB network, as it does not readily form pairs or aggregates.

There is another interesting observation beyond the formation of aggregates. KSCN and KSeCN, despite having different polarizabilities, show similar behaviors in several aspects, e.g., their effects on water HB, refractive index, viscosity, Jones–Dole *B* coefficients, standard partial molar volumes, and conductivity. This similarity is particularly odd for KSeCN, given its higher polarizability, which would typically suggest a more chaotropic behavior based on the recalled generic definitions. This highlights the importance of the anisotropic nature of polarizability in polyatomic ions, as it ultimately determines the physicochemical properties of their solutions.

While the aggregate formation plays a very important role in the investigated properties, it cannot account for the entire set of observations. The behavior of KSeCN is unique and appears to be linked to its electronic structure. This could be explained by considering an uneven charge distribution that leads to an unusual interaction with water molecules, similar to what was found in the case of iodate [27]. The larger polarizability of selenium can justify a redistribution of its electronic cloud, which in turn may result in a lowering of the partial charge located on Se and in a change in the anion hydration properties. While this hypothesis aligns with the observed trends, further studies are needed to fully understand the underlying mechanism and charge distribution, including a computational investigation.

## 4. Materials and Methods

### 4.1. Chemicals

Ultra-pure Milli-Q water from Millipore with a resistivity of 18.2 MΩ∙cm and conductivity of 0.055 μS∙cm^−1^ was used. Potassium cyanate (KOCN), potassium thiocyanate (KSCN), and potassium selenocyanate (KSeCN) were purchased from Merck (Milan, Italy) with stated purities of 97%, 99+%, and 99+%, respectively, and used without further purification. The solutions were prepared by weighing the required amounts of salts and water. Molal concentrations were converted into molar units using the measured density values.

### 4.2. Methods

Methods used are described below. For the calculation of various physicochemical parameters, such as apparent molar volumes, partial molar volumes, Jones–Dole equation coefficients, polarizability, and limiting conductivities, a detailed description is available in Appendix A of a previous work [27].

#### 4.2.1. Conductivity

Conductivity measurements were made using a Hach senIonTM+ EC7 conductivity meter (Lainate, Italy) with an error margin of less than 0.1% for conductivity and 0.2% for temperature. Two probes were employed to account for the wide range of conductivity values between Milli-Q water and salt solutions. The probes used were sensIonTM+ 50 70 (range: 0.2 μS/cm to 200 mS/cm) and sensIonTM+ 50 71 (range: 0.05 μS/cm to 30 mS/cm). All conductivity measurements were performed at 25 °C.

The limiting molar conductivity (*Λ*^∞^, in S·cm^2^·mol^−1^) was extrapolated according to the modified Fuoss–Hsia equation, Equation (2) [70,71]:

(1) $$\Lambda =\Lambda^{\infty }-S\sqrt{c}+Ec\cdot ln\left(c\right)+J_{1}c-J_{2}c^{3/2}$$

where *S* and *E* are related to the charge, mobility of the ions, and to the dielectric constant and viscosity of the solvent. *J*_1_ and *J*_2_ are specific to each electrolyte. This equation assumes a complete dissociation of the salts and therefore was used to fit *Λ* for c < 1 M.

#### 4.2.2. Density

Density measurements were performed with an accuracy of ±5∙10^−6^ g∙cm^−3^ using an Anton-Paar© DMA 5000 density meter. Measurements were conducted at 25 °C with a temperature precision of ±0.001 °C as a function of salt concentration.

The obtained values were used to calculate the apparent molar volume, the standard partial molar volume, and the partial molar volumes of the solutions.

The apparent molar volumes (Equation (2)) and partial molar volumes (Equation (3)) were calculated according to the work of Millero [72]:

(2) V¯2ϕ=1000ρ*−ρm2ρρ*+M2ρ

where V¯2ϕ is the apparent molar volume, ρ* and *ρ* are the densities of the pure solvent and of the solution at the same temperature, *m*_2_ is the solute concentration in molal units and *M*_2_ is the molar mass of the solute.

(3) V¯2=V¯2Φ+1000−cΦV¯22000+cc∂ΦV¯2∂cT,P,n1∂ΦV¯2∂cT,P,n1c

where ${\bar{V}}_{2}$ is the partial molar volume and *c* is the molar concentration.

Apparent molar volumes were fitted according to the extended Redlich, Rosenfeld, and Meyer’s (RRM) model to extract the standard partial molar volumes [72,73]:

(4) V¯2Φ=V¯20+ac2+bVc2

#### 4.2.3. Viscosity

Viscosity measurements were conducted at 25 °C as a function of the salt concentration using a Ubbelohde viscometer from Schott (Mainz, Germany) with a capillary diameter of 0.36 ± 0.01 mm. The water bath was maintained by a Lauda E200 thermostat with a Pt-100 temperature probe with a temperature accuracy of ±0.01 °C. Solutions were equilibrated in the water bath for 30 min before measurements. Flow times were converted to viscosity (in mPa·s) using the formula *η* = *Aρt*, where *ρ* is the density of the investigated solution. The instrument constant *A* was calculated using the tabulated viscosity value for pure water at 25 °C (0.89040 mPa·s).

Viscosity values were fitted with the Jones–Dole equation (see Equation (5)) [46,47]. This semi-empirical equation describes the relative viscosity of electrolyte solutions as a function of concentration.

(5) $$\left(\frac{\eta }{\eta_{0}}\right)-1=A\sqrt{c}+Bc+Dc^{2}$$

where *η* and *η*_0_ are the viscosities of the solution and of the pure solvent at the same temperature, respectively.

#### 4.2.4. Attenuated Total Reflection Fourier Transformed Infrared Spectroscopy

Fourier-transform infrared spectroscopy (FTIR) was utilized to analyze aqueous solutions of each salt in attenuated total reflection (ATR) mode using a Thermo Nicolet Nexus 870 instrument (Monza, Italy) with an MCT (mercury cadmium telluride) detector. The spectra were recorded within a range of 650 to 4000 cm^−1^, at a resolution of 2 cm^−1^, and with 128 scans for each spectrum. All data were normalized using standard normal variate (SNV) normalization, with offset normalization applied to adjust for baseline shifts and intensity variations, ensuring that the absorbance was at a minimum of zero. A Savitsky–Golay smoothing function with a polynomial order of 2 was used, incorporating a seven-point smoothing process.

Deconvolution of the spectra was performed on the -OH stretching bands of water with a four-component Gaussian model [49,50,51]. The deconvoluted water bands were then assigned to different types according to the literature [49,50,51].

#### 4.2.5. Refractive Index

Refractive index measurements, accurate to ±0.0002 nD, were conducted at 20 °C using an Abbé refractometer (model NAR-1T LIQUID, Atago Italia Srl, Milan, Italy). The refractometer was connected to a water bath to maintain a stable temperature during the measurements.

The refractive index of salts (*n*_salt_) was calculated according to the De Feijter equation [74,75]:

(6) $$\frac{dn}{dc}=\frac{1}{\rho_{salt\;}}\left(n_{salt\;}-n_{water\;}\right)$$

where *n* is the refractive index and *ρ*_salt_ is the density of pure salt. The polarizabilities (*α*) were then calculated by the Lorentz–Lorenz equation [74,76]:

(7) $$\alpha_{salt}=\frac{3}{4\pi N_{A}}\left(\frac{n_{salt}^{2}-1}{n_{salt}^{2}+2}\right)V_{m}$$

where *N*_A_ is the Avogadro number and *V*_m_ is the molar volume of the salt.

#### 4.2.6. Debye and Bjerrum Length

The Debye and Bjerrum lengths describe the scale at which electrostatic interactions become relevant in a solution. The Debye length is the distance over which charge screening occurs due to the presence of mobile ions [77]. On the other hand, the Bjerrum length is the distance at which electrostatic interactions between two charged particles are comparable to thermal energy [77]. These lengths help characterize the behavior of electrolyte solutions, e.g., ion pairing.

The Debye length was calculated according to Equation (8):

(8) $$\lambda_{D}=\sqrt{\frac{\varepsilon_{0}\varepsilon_{r}k_{B}T}{1000\cdot N_{A}e^{2}\sum c_{i}z_{i}^{2}}}$$

and the Bjerrum length was calculated according to Equation (9):

(9) $$\lambda_{B}=\frac{e^{2}}{4\pi \varepsilon_{0}\varepsilon_{r}k_{B}T}$$

where *ε*_0_ (in F·m^−2^) is the vacuum permittivity, *ε*_r_ is the relative permittivity of the solvent, *k*_B_ (in J·K^−1^) is the Boltzmann constant, *T* (in K) is the absolute temperature, *N*_A_ (mol^−1^) is the Avogadro number, *e* is the elementary charge (in C), *c* (in M) is the concentration, and *z* is the ion charge.

## 5. Conclusions

In the present study, we investigated the complexity of specific ion effects in potassium pseudohalide salts (KOCN, KSCN, KSeCN) through experimental measurements of conductivity, density, viscosity, infrared spectra, and refractive index as a function of the electrolyte concentration at constant temperature (25 °C).

We demonstrated that traditional frameworks for ion classification based on polarizability fail to fully capture the behavior of these polyatomic ions. The polarizability of isoelectronic polyatomic ions does indeed influence their ion-specific behavior. But it is the anisotropic nature of this polarizability, along with the ion’s shape and electronic structure, that plays a decisive role in determining their hydration, aggregation, solute–solute, and solute–solvent interactions. For instance, KSeCN exhibits a less chaotropic behavior than expected. This behavior is reminiscent of that of iodate ions that act as a typical kosmotrope. In conclusion, we show that in the case of these isoelectronic ions XCN^−^ (where X = O, S, or Se), the overall nature of the ion is not simply a function of the polarizability, but it depends on the specific local interactions with the first layers of solvation.

We also reported that KSCN and KSeCN tend to form ion pairs and higher-order aggregates, a behavior commonly observed in aprotic polar solvents.

Future studies will certainly provide valuable insights into these processes and help improve our understanding of this specific ion phenomenon.

## Figures and Tables

**Table 1:** Partial charges on the constituent atoms of XCN^−^. Here, X = O, S, or Se in cyanate, thiocyanate, and selenocyanate, respectively.

	Partial Charges					
Ion	X		C		N	
OCN^−^	−0.514 ^a^	−0.54 ^b^	−0.019 ^a^	0.21 ^b^	−0.467 ^a^	−0.67 ^b^
SCN^−^	−0.577 ^a^	−0.56 ^b^	−0.187 ^a^	−0.15 ^b^	−0.236 ^a^	−0.30 ^b^
SeCN^−^	/	−0.53 ^b^	/	−0.28 ^b^	/	−0.19 ^b^

^a^: from ref. [33]; ^b^: calculated with MolCalc [34].

**Table 2:** Extrapolated limiting molar conductivity values *Λ*^∞^ in S·cm^2^·mol^−1^. The data obtained in this work compared to those extracted from the literature [37] for each salt at 25 °C.

Salt	This Work	From the Literature
KOCN	138.0 ± 0.2	138.1
KSCN	139.9 ± 0.2	139.5
KSeCN	138.6 ± 0.2	138.2

**Table 3:** Standard partial molar volumes ${\bar{V}}_{2}^{o}$ in cm^3^·mol^−1^ calculated according to Equation (4) and compared to the literature values for KOCN, KSCN and KSeCN at 25 °C.

Salt	This Work	From the Literature
KOCN	33.8 ± 0.4	35.1 ^a^
KSCN	49.5 ± 0.1	49.0 ^b^
KSeCN	53.3 ± 0.9	58.7 ^a^

^a^: from ref. [39]; ^b^: from tef. [38].

**Table 4:** *A* (in L^1/2^·mol^−1/2^), *B*_JD_ (in L·mol^−1^), and *D* (in L^3/2^·mol^−3/2^) coefficients obtained by fitting the viscosity data with Equation (5).

Salt	*A*	*B*	*D*
KOCN	0.0056	0.009 ± 0.002	0.016 ± 0.001
KSCN	0.0056	−0.047 ± 0.001	0.016 ± 0.001
KSeCN	0.0055	−0.048 ± 0.002	0.019 ± 0.001

**Table 5:** Refractive index (*n*) of the salts and polarizability (*α*, in Å^3^) at 25 °C calculated according to Equations (6) and (7), respectively.

Salt	*n*	*α*
KOCN	1.551 ± 0.004	4.98 ± 0.02
KSCN	1.652 ± 0.008	7.41 ± 0.02
KSeCN	1.692 ± 0.005	9.32 ± 0.03

## Data Availability

The data supporting reported results can be found with the authors.

## Supplementary Materials

The following supporting information can be downloaded at: https://www.mdpi.com/article/10.3390/molecules30020323/s1, Figure S1: Conductivity, *κ* as a function of the salt concentration (*c*, in molal units) for potassium cyanate (black), thiocyanate (blue) and selenocyanate (red) solutions at 25° C. Open circles show values taken from ref 1.; Figure S2: Apparent molar volume V2ϕ as a function of the salt concentration (*c*, in molal units) for potassium cyanate (black), thiocyanate (blue) and selenocyanate (red) solutions at 25 °C; Figure S3: Infrared absorbance spectra of (a) KOCN, (b) KSCN and (c) KSeCN solutions. The spectra were truncated to the CN stretching region and normalized to the maximum of absorbance value. The salt concentrations in each spectrum are 0.5 (━), 1 (━), 1.5 (━), 2 (━), 2.5 (━) m. The dashed band is the spectrum of pure water. The red arrow indicates the red shift of the absorption band with increasing concentration; Figure S4: Infrared absorbance spectra of (a) KOCN, (b) KSCN and (c) KSeCN solutions. The spectra were truncated to the OH bending region and normalized to the maximum of absorbance value. The salt concentrations in each spectrum are 0.5 (━), 1 (━), 1.5 (━), 2 (━), 2.5 (━) m. The dashed band is the spectrum of pure water. The red arrow indicates the red shift of the absorption band with increasing concentration; Figure S5: Infrared absorbance spectra of (a) KOCN, (b) KSCN and (c) KSeCN solutions. The spectra were truncated to the OH stretching region and normalized to the maximum of absorbance value. The salt concentrations in each spectrum are 0.5 (━), 1 (━), 1.5 (━), 2 (━), 2.5 (━) m. The dashed band is the spectrum of pure water. The blue arrows indicate the blue shift of the absorption bands of two different kinds of water with increasing concentration; Figure S6: Deconvolution of IR absorbance spectra of pure water. The spectrum was truncated to the OH stretching region and normalized to the maximum of absorbance. Four different kinds of water contributions Type I (━), Type II (━), Type III (━) and Type IV (━) are shown. Black line is the original spectrum and green dotted line is the fitting. The residuals of the fitting are shown on top; Figure S7: Deconvolution of IR absorbance spectra of KOCN at (a) 0.5, (b) 1, (c) 1.5, (d) 2 and (e) 2.5 m. The spectrum was truncated to the OH stretching region and normalized to the maximum of absorbance value. Four different kinds of water contributions Type I (━), Type II (━), Type III (━) and Type IV (━) are shown. Black line is the original spectrum and green dotted line is the fitting. The residuals of the fitting are shown on top; Figure S8: Deconvolution of IR absorbance spectra of KSCN at (a) 0.5, (b) 1, (c) 1.5, (d) 2 and (e) 2.5 m. The spectrum was truncated to the OH stretching region and normalized to the maximum of absorbance value. Four different kinds of water contributions Type I (━), Type II (━), Type III (━) and Type IV (━) are shown. Black line is the original spectrum and green dotted line is the fitting. The residuals of the fitting are shown on top; Figure S9: Deconvolution of IR absorbance spectra of KSeCN at (a) 0.5, (b) 1, (c) 1.5, (d) 2 and (e) 2.5 m. The spectrum was truncated to the OH stretching region and normalized to the maximum of absorbance value. Four different kinds of water contributions Type I (━), Type II (━), Type III (━) and Type IV (━) are shown. Black line is the original spectrum and green dotted line is the fitting. The residuals of the fitting are shown on top; Table S1: Conductivity (*κ*, in mS·cm^−1^) and molar conductivity (*Λ*, in S·cm^2^·mol^−1^) values at 25 °C of potassium cyanate, thiocyanate and selenocyanate solutions at different concentrations (*c*, in molar units). The experimental errors on *κ* and on *Λ* are ±0.1% and ±0.1, respectively; Table S2: Density values (*ρ*, in g·cm^−3^) at 25 °C of potassium cyanate, thiocyanate and selenocyanate aqueous solutions at different concentrations (*c*, in molar units). The experimental errors are ±1 × 10^−5^; Table S3: Apparent molar (V2ϕ, in cm^3^∙mol^−1^) and partial molar volumes ($\bar{V_{2}}$, in cm^3^∙mol^−1^) at 25 °C of potassium cyanate, thiocyanate and selenocyanate solutions at different concentrations (*c*, in molar units). The experimental errors are ±1.3; Table S4: Viscosity (*η*, in mPa·s) of potassium cyanate, thiocyanate and selenocyanate aqueous solutions at different molar concentrations *c* at 25 °C. The experimental errors are ±1 × 10^−3^; Table S5: Refractive index (*n*) at 25 °C of potassium cyanate, thiocyanate and selenocyanate aqueous solutions at different molar concentrations *c* at 25 °C. The experimental errors are ±1 × 10^−4^.
