# Supplementary material for: The Role of Polarizability in Isoelectronic Ions: The Case of Pseudohalides
Source: Molecules. 2025 Jan 15;30(2):323. doi: 10.3390/molecules30020323 (PMC11767347; doi:10.3390/molecules30020323)
Supplement: Supplementary file 1 [file molecules-30-00323-s001.zip › molecules-3398600-supplementary.pdf]

Supporting Material

The Role of Polarizability in Isoelectronic Ions: The Case of Pseudohalides

Mert Acar[1], Duccio Tatini[2], Barry W. Ninham[3] and Pierandrea Lo Nostro[1,*]

[1] Department of Chemistry "Ugo Schiff" and CSGI, University of Florence, 50019 Sesto Fiorentino, Italy; mert.acar@unifi.it
[2] Department of Biotechnologies, Chemistry and Pharmacy, University of Siena, 53100 Siena, Italy; duccio.tatini@unisi.it
[3] Materials Physics (Formerly Department of Applied Mathematics), Research School of Physics, Australian National University, Canberra, ACT 2600, Australia
* Correspondence: pierandrea.lonostro@unifi.it; Tel.: +39-055-4573010

Supporting Figures

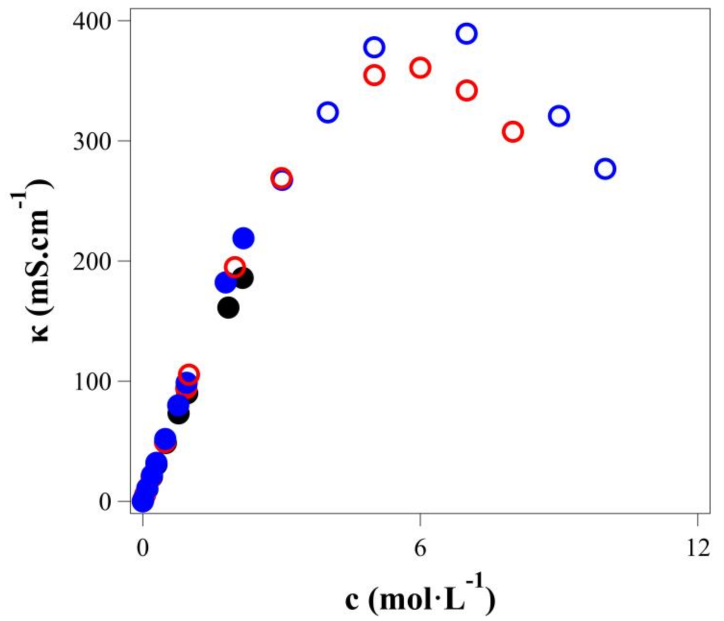

Figure S1. Conductivity, κ as a function of the salt concentration (c, in molal units) for potassium cyanate (black), thiocyanate (blue) and selenocyanate (red) solutions at 25° C. Open circles show values taken from ref 1.

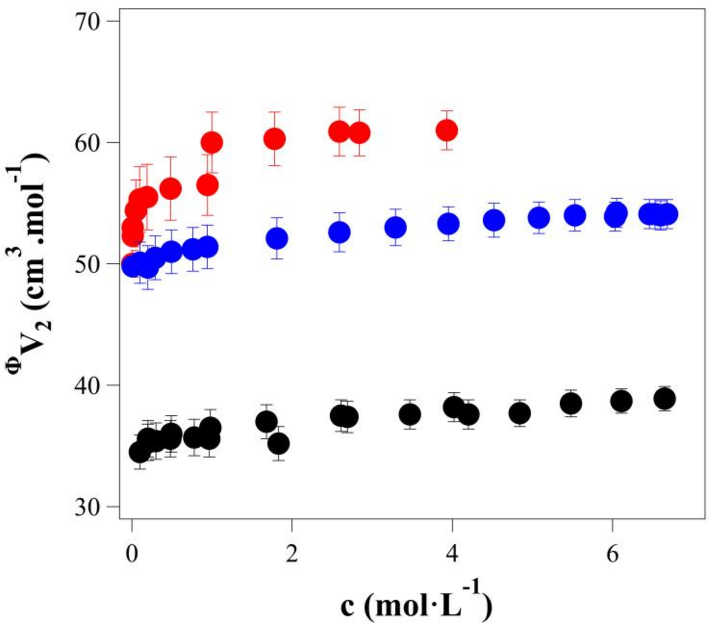

Figure S2. Apparent molar volume $^{\phi}V_2$ as a function of the salt concentration (*c*, in molal units) for potassium cyanate (black), thiocyanate (blue) and selenocyanate (red) solutions at 25° C.

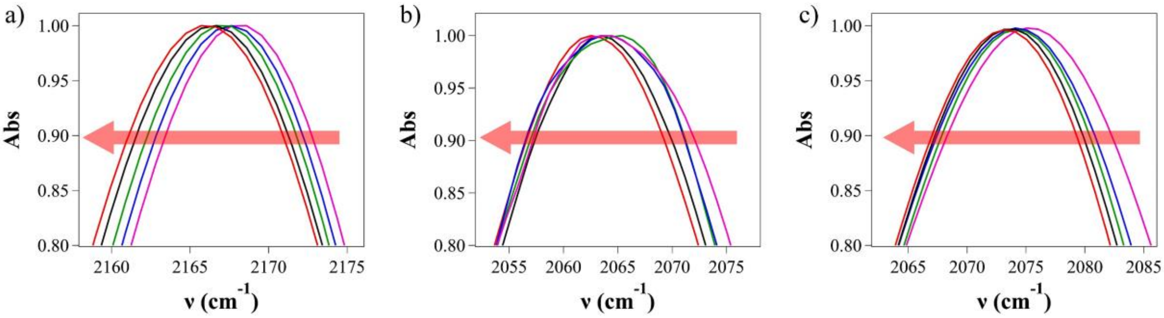

Figure S3. Infrared absorbance spectra of (a) KOCN, (b) KSCN and (c) KSeCN solutions. The spectra were truncated to the CN stretching region and normalized to the maximum of absorbance value. The salt concentrations in each spectrum are 0.5 (—), 1 (—), 1.5 (—), 2 (—), 2.5 (—) m. The dashed band is the spectrum of pure water. The red arrow indicates the red shift of the absorption band with increasing concentration.

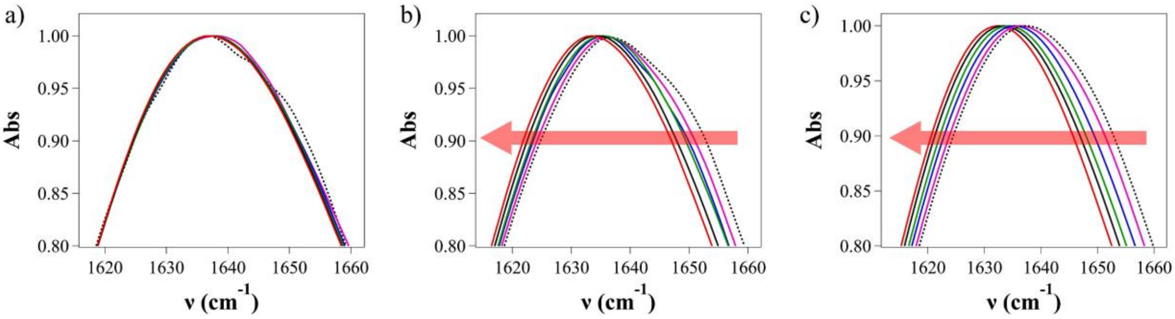

Figure S4. Infrared absorbance spectra of (a) KOCN, (b) KSCN and (c) KSeCN solutions. The spectra were truncated to the OH bending region and normalized to the maximum of absorbance value. The salt concentrations in each spectrum are 0.5 (—), 1 (—), 1.5 (—), 2 (—

), 2.5 (—) m. The dashed band is the spectrum of pure water. The red arrow indicates the red shift of the absorption band with increasing concentration.

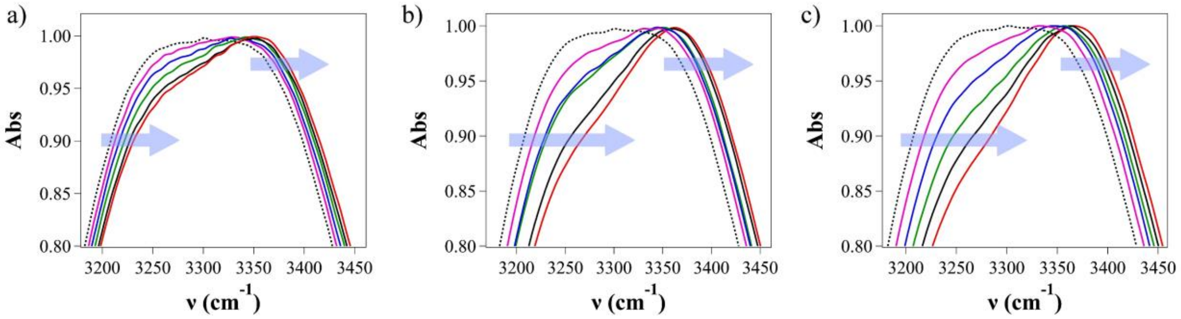

Figure S5. Infrared absorbance spectra of (a) KOCN, (b) KSCN and (c) KSeCN solutions. The spectra were truncated to the OH stretching region and normalized to the maximum of absorbance value. The salt concentrations in each spectrum are 0.5 (—), 1 (—), 1.5 (—), 2 (—), 2.5 (—) m. The dashed band is the spectrum of pure water. The blue arrows indicate the blue shift of the absorption bands of two different kinds of water with increasing concentration.

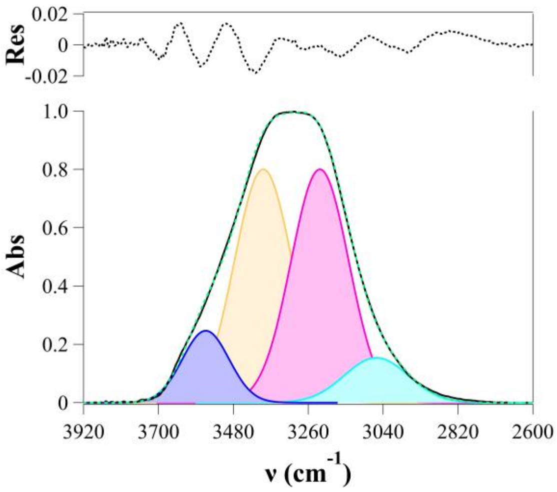

Figure S6. Deconvolution of IR absorbance spectra of pure water. The spectrum was truncated to the OH stretching region and normalized to the maximum of absorbance. Four different kinds of water contributions Type I (—), Type II (—), Type III (—) and Type IV (—) are shown. Black line is the original spectrum and green dotted line is the fitting. The residuals of the fitting are shown on top.

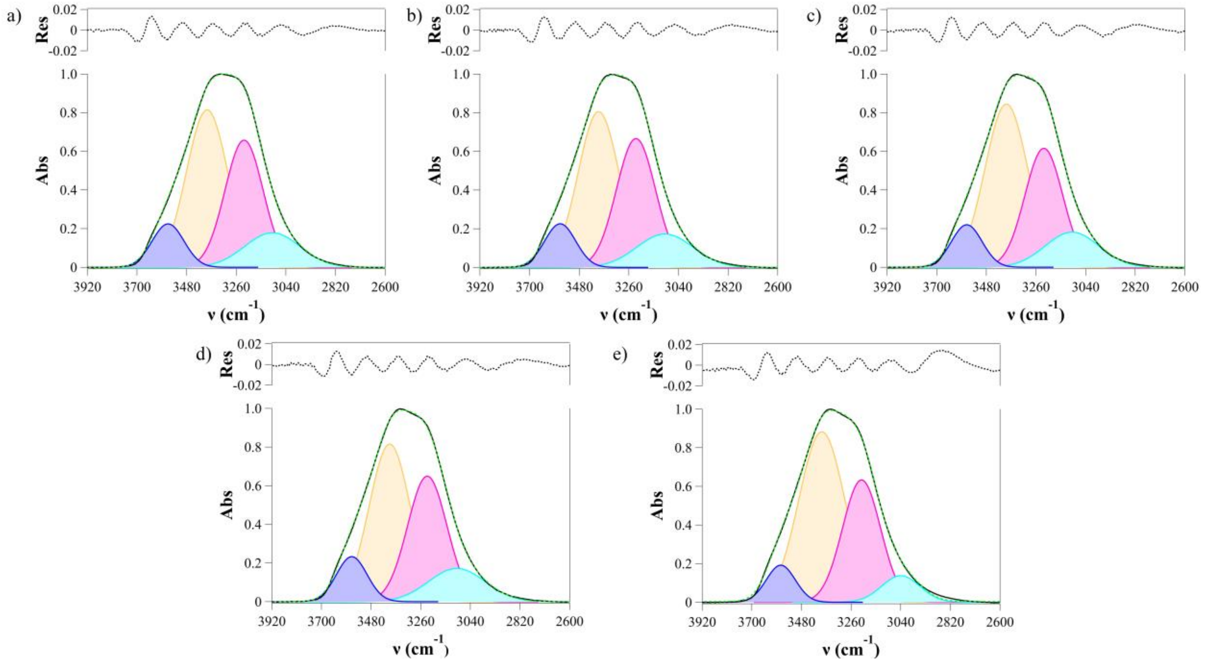

Figure S7. Deconvolution of IR absorbance spectra of KOCN at (a) 0.5, (b) 1, (c) 1.5, (d) 2 and (e) 2.5 m. The spectrum was truncated to the OH stretching region and normalized to the maximum of absorbance value. Four different kinds of water contributions Type I (—), Type II (—), Type III (—) and Type IV (—) are shown. Black line is the original spectrum and green dotted line is the fitting. The residuals of the fitting are shown on top.

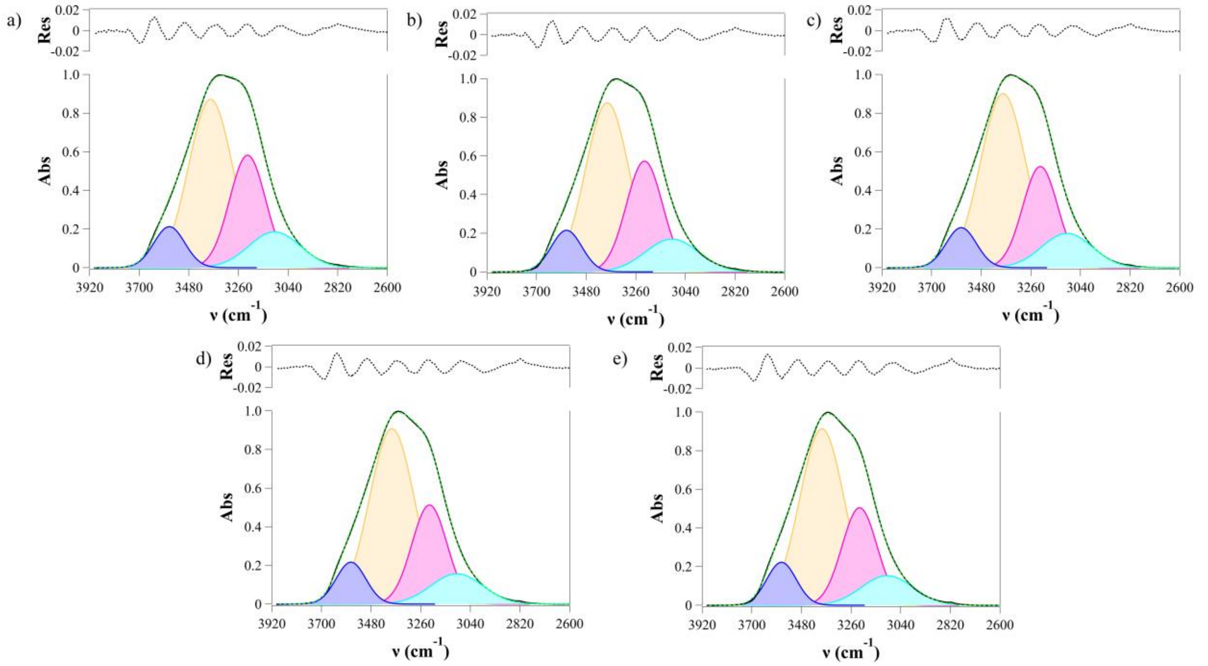

Figure S8. Deconvolution of IR absorbance spectra of KSCN at (a) 0.5, (b) 1, (c) 1.5, (d) 2 and (e) 2.5 m. The spectrum was truncated to the OH stretching region and normalized to the maximum of absorbance value. Four different kinds of water contributions Type I (—), Type II (—), Type III (—) and Type IV (—) are shown. Black line is the original spectrum and green dotted line is the fitting. The residuals of the fitting are shown on top.

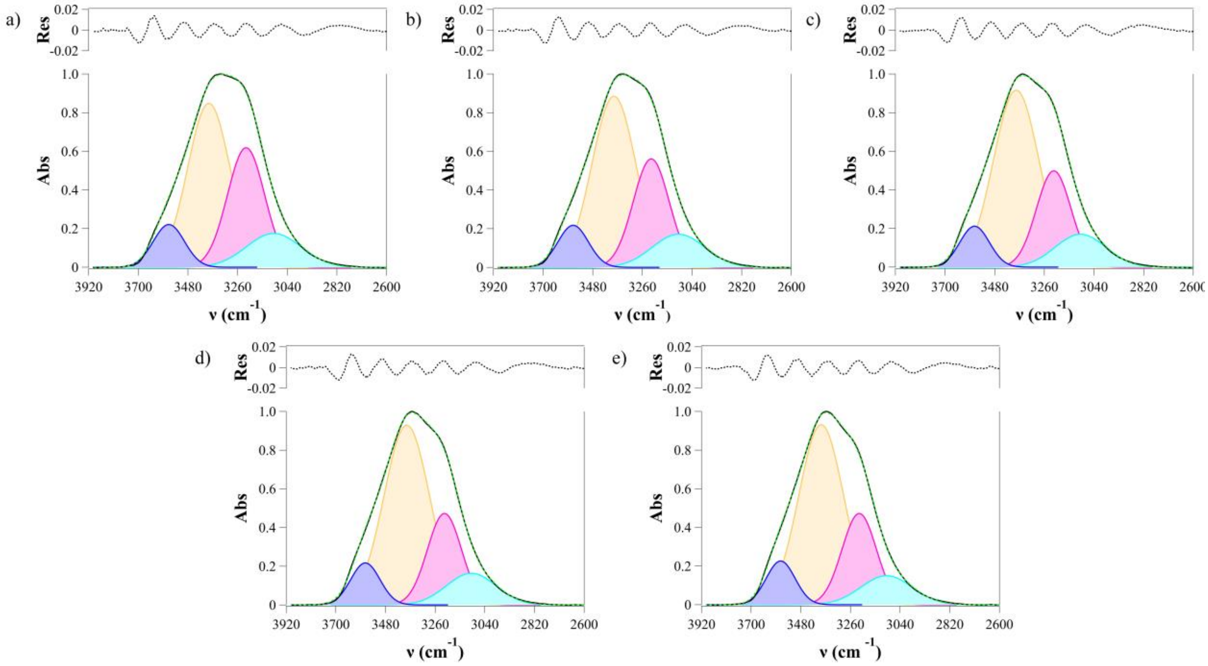

Figure S9. Deconvolution of IR absorbance spectra of KSeCN at (a) 0.5, (b) 1, (c) 1.5, (d) 2 and (e) 2.5 m. The spectrum was truncated to the OH stretching region and normalized to the maximum of absorbance value. Four different kinds of water contributions Type I (—), Type II (—), Type III (—) and Type IV (—) are shown. Black line is the original spectrum and green dotted line is the fitting. The residuals of the fitting are shown on top.

Supporting Tables

Table S1. Conductivity (κ, in mS·cm^{-1}) and molar conductivity (Λ, in S·cm^{2}·mol^{-1}) values at 25 °C of potassium cyanate, thiocyanate and selenocyanate solutions at different concentrations (c, in molar units). The experimental errors on κ and on Λ are ±0.1% and ±0.1, respectively.

KOCN			KSCN			KSeCN		
c	κ	Λ	c	κ	Λ	c	κ	Λ
$9.94\cdot10^{-4}$	0.126	127.4	$1.00\cdot10^{-3}$	0.129	128.4	$9.95\cdot10^{-4}$	0.129	130.0
$1.00\cdot10^{-2}$	1.13	112.6	$9.98\cdot10^{-3}$	1.32	131.9	$4.99\cdot10^{-3}$	0.638	127.9
$9.95\cdot10^{-2}$	10.3	103.2	$9.89\cdot10^{-2}$	10.9	110.3	$1.00\cdot10^{-2}$	1.27	126.8
$1.99\cdot10^{-1}$	20.4	102.8	$1.98\cdot10^{-1}$	21.7	109.8	$4.94\cdot10^{-2}$	5.57	112.7
$2.96\cdot10^{-1}$	30.7	103.8	$2.94\cdot10^{-1}$	32.1	109.0	$1.89\cdot10^{-1}$	20.7	109.5
$4.97\cdot10^{-1}$	48.6	97.7	$4.87\cdot10^{-1}$	51.9	106.6	$4.81\cdot10^{-1}$	49.3	102.5
$7.73\cdot10^{-1}$	73.2	94.7	$7.65\cdot10^{-1}$	79.9	104.4	$9.39\cdot10^{-1}$	94.2	100.3
$9.60\cdot10^{-1}$	90.0	93.7	$9.49\cdot10^{-1}$	98.7	104.0			
1.85	161	87.2	1.79	182	101.6			
2.16	186	86.2	2.18	219	100.6			

Table S2. Density values (ρ, in g·cm^{-3}) at 25 °C of potassium cyanate, thiocyanate and selenocyanate aqueous solutions at different concentrations (c, in molar units). The experimental errors are ±1·10^{-5}.

KOCN		KSCN		KSeCN	
c	*ρ*	*c*	*ρ*	*c*	*ρ*
0	0.99703	0	0.99703	0	0.99703
$1.00\cdot10^{-3}$	0.99709	$9.99\cdot10^{-4}$	0.99708	$9.95\cdot10^{-4}$	0.99712
$9.67\cdot10^{-3}$	0.99750	$9.89\cdot10^{-3}$	0.99747	$4.99\cdot10^{-3}$	0.99747
$9.96\cdot10^{-2}$	1.0017	$9.90\cdot10^{-2}$	1.0017	$4.40\cdot10^{-3}$	0.99743
$1.98\cdot10^{-1}$	1.0061	$1.98\cdot10^{-1}$	1.0064	$8.98\cdot10^{-3}$	0.99782
$3.00\cdot10^{-1}$	1.0107	$2.90\cdot10^{-1}$	1.0106	$1.00\cdot10^{-2}$	0.99792
$4.79\cdot10^{-1}$	1.0189	$4.93\cdot10^{-1}$	1.0198	$4.94\cdot10^{-2}$	1.0014
$7.78\cdot10^{-1}$	1.0324	$7.61\cdot10^{-1}$	1.0321	$9.62\cdot10^{-2}$	1.0056
$9.66\cdot10^{-1}$	1.0411	$9.42\cdot10^{-1}$	1.0402	$1.89\cdot10^{-1}$	1.0138
1.83	1.0814	1.81	1.0788	$4.82\cdot10^{-1}$	1.0394
2.69	1.1148	2.59	1.1131	$9.44\cdot10^{-1}$	1.0798
3.47	1.1483	3.29	1.1428		
4.20	1.1802	3.95	1.1709		
4.84	1.2074	4.52	1.1947		
5.48	1.2311	5.08	1.2184		
6.11	1.2568	5.53	1.2367		
6.65	1.2784	6.05	1.2581		

Table S3. Apparent molar (${}^{\phi}V_2$, in $cm^3\cdot mol^{-1}$) and partial molar volumes ($\overline{V_2}$, in $cm^3\cdot mol^{-1}$) at 25 °C of potassium cyanate, thiocyanate and selenocyanate solutions at different concentrations (*c*, in molar units). The experimental errors are ±1.3.

KOCN			KSCN			KSeCN		
c	${}^{\phi}V_2$	$\overline{V_2}$	*c*	${}^{\phi}V_2$	$\overline{V_2}$	*c*	${}^{\phi}V_2$	$\overline{V_2}$
$9.67\cdot10^{-3}$	29.5	30.6	9.89·10-3	49.8	49.9	$4.99\cdot10^{-3}$	50.0	52.6
$9.96\cdot10^{-2}$	34.5	37.0	9.90·10-2	50.1	50.1	$4.40\cdot10^{-3}$	46.4	48.2
$1.98\cdot10^{-1}$	35.3	37.1	1.98·10-1	49.7	50.1	$8.98\cdot10^{-3}$	53.0	55.3
$2.01\cdot10^{-1}$	35.6[a]	35.8[a]	4.93·10-1	51.0	51.7	$1.00\cdot10^{-2}$	52.3	53.7
$3.00\cdot10^{-1}$	35.4	35.4	7.61·10-1	51.2	51.8	$4.94\cdot10^{-2}$	54.4	55.5
$4.79\cdot10^{-1}$	35.6	36.9	9.42·10-1	51.4	52.3	$9.62\cdot10^{-2}$	55.3	56.1
$4.91\cdot10^{-1}$	36.0[a]	36.2[a]	1.81	52.1	53.3	$1.89\cdot10^{-1}$	55.5	61.7
$7.78\cdot10^{-1}$	35.7	36.4	2.59	52.6	54.1	$1.99\cdot10^{-1}$	59.4[a]	60.0[a]
$9.79\cdot10^{-1}$	36.5[a]	36.0[a]	3.29	53.0	54.6	$4.82\cdot10^{-1}$	56.2	54.4
$9.66\cdot10^{-1}$	35.6	36.4	3.95	53.3	55.1	$9.44\cdot10^{-1}$	56.5	62.2
1.68	37.0[a]	36.3[a]	4.52	53.6	55.5	1.78	60.3[a]	61.2[a]
1.83	35.2	36.2	5.08	53.8	55.7	2.59	60.9[a]	62.0[a]
2.61	37.5	43.4	5.53	54.0	56.1	2.84	60.8[a]	61.0[a]
2.69	37.4	37.7	6.05	54.2	53.1	3.93	61.0[a]	61.6[a]
4.02	38.2	38.2	6.46	53.9[b]	52.5[b]			
4.20	37.6	35.0	6.53	54.1[b]	56.5[b]			
4.84	37.7	40.9						
5.48	38.5	42.5						
6.11	38.7	40.7						
6.65	38.9	41.2						

[a] Calculated from ref. 2.
[b] Calculated from ref. 3.

Table S4. Viscosity (η, in mPa·s) of potassium cyanate, thiocyanate and selenocyanate aqueous solutions at different molar concentrations c at 25 °C. The experimental errors are $\pm 1\cdot 10^{-3}$.

KOCN		KSCN		KSeCN	
c	η	c	η	c	η
0	0.890	0	0.890	0	0.890
$9.97\cdot 10^{-3}$	0.894	$1.00\cdot 10^{-2}$	0.891	$9.91\cdot 10^{-4}$	0.893
$4.92\cdot 10^{-2}$	0.891	$4.97\cdot 10^{-2}$	0.890	$4.99\cdot 10^{-3}$	0.893
$9.94\cdot 10^{-2}$	0.892	$9.88\cdot 10^{-2}$	0.889	$9.96\cdot 10^{-3}$	0.893
$1.97\cdot 10^{-1}$	0.894	$1.97\cdot 10^{-1}$	0.885	$5.30\cdot 10^{-2}$	0.893
$2.96\cdot 10^{-1}$	0.895	$3.88\cdot 10^{-1}$	0.879	$1.05\cdot 10^{-1}$	0.890
$4.86\cdot 10^{-1}$	0.901	$7.61\cdot 10^{-1}$	0.873	$2.09\cdot 10^{-1}$	0.887
$7.67\cdot 10^{-1}$	0.911	$9.48\cdot 10^{-1}$	0.869	$5.17\cdot 10^{-1}$	0.881
$9.67\cdot 10^{-1}$	0.917	1.13	0.866	1.00	0.869
1.85	0.959	1.47	0.867	1.78	0.872
2.69	1.015	1.64	0.867	2.58	0.901
3.45	1.092	1.81	0.869	2.83	0.909
4.20	1.187	2.60	0.886	3.92	0.992
4.76	1.268	3.31	0.917		
5.51	1.369	3.98	0.959		
6.08	1.462	4.56	1.005		
6.59	1.583	5.15	1.067		

Table S5. Refractive index (n) at 25 °C of potassium cyanate, thiocyanate and selenocyanate aqueous solutions at different molar concentrations c at 25 °C. The experimental errors are $\pm 1\cdot 10^{-4}$.

KOCN		KSCN		KSeCN	
c	n	c	n	c	n
0	1.3325	0	1.3325	0	1.3325
$8.08\cdot 10^{-4}$	1.3328	$9.69\cdot 10^{-4}$	1.3325	$1.43\cdot 10^{-4}$	1.3325
$4.03\cdot 10^{-3}$	1.3330	$4.84\cdot 10^{-3}$	1.3330	$7.19\cdot 10^{-4}$	1.3325
$8.04\cdot 10^{-3}$	1.3333	$9.64\cdot 10^{-3}$	1.3335	$1.44\cdot 10^{-4}$	1.3327
$1.56\cdot 10^{-2}$	1.3341	$1.34\cdot 10^{-2}$	1.3345	$6.33\cdot 10^{-4}$	1.3325
$2.41\cdot 10^{-2}$	1.3350	$1.92\cdot 10^{-2}$	1.3353	$1.29\cdot 10^{-3}$	1.3327
$3.97\cdot 10^{-2}$	1.3369	$2.86\cdot 10^{-2}$	1.3370	$7.12\cdot 10^{-3}$	1.3339
$6.23\cdot 10^{-2}$	1.3392	$4.83\cdot 10^{-3}$	1.3327	$1.39\cdot 10^{-2}$	1.3348
$7.48\cdot 10^{-2}$	1.3404	$9.60\cdot 10^{-3}$	1.3334	$2.72\cdot 10^{-2}$	1.3370
$1.51\cdot 10^{-1}$	1.3480	$3.77\cdot 10^{-2}$	1.3381	$6.93\cdot 10^{-2}$	1.3430
$2.17\cdot 10^{-1}$	1.3543	$5.67\cdot 10^{-2}$	1.3420	$1.35\cdot 10^{-1}$	1.3532
$2.78\cdot 10^{-1}$	1.3602	$7.68\cdot 10^{-2}$	1.3450		
$3.38\cdot 10^{-1}$	1.3662	$8.97\cdot 10^{-2}$	1.3476		
$3.835\cdot 10^{-1}$	1.3702	$1.146\cdot 10^{-1}$	1.3523		

KOCN		KSCN		KSeCN	
c	*n*	*c*	*n*	*c*	*n*
$4.439 \cdot 10^{-1}$	1.3750	$1.353 \cdot 10^{-1}$	1.3560		
$4.913 \cdot 10^{-1}$	1.3786	$1.543 \cdot 10^{-1}$	1.3596		
$5.331 \cdot 10^{-1}$	1.3816	$1.773 \cdot 10^{-1}$	1.3636		
		$2.154 \cdot 10^{-1}$	1.3701		
		$2.517 \cdot 10^{-1}$	1.3766		

References.

1. Bujewska, P.; Gorska, B.; Fic, K. Redox Activity of Selenocyanate Anion in Electrochemical Capacitor Application. *Synthetic Metals* **2019**, *253*, 62-72, doi:10.1016/j.synthmet.2019.04.024.

2. Maurey, J.R.; Wolff, J. The Partial Molal Volumes of OCN^-, $SeCN^-$, ReO_4^-, BF_4^-, SO_3F^-, $SO_3NH_2^-$. *Journal of Inorganic and Nuclear Chemistry* **1963**, *25*, 312-314, doi:10.1016/0022-1902(63)80062-7.

3. Mitchell, J.P.; Butler, J.B.; Albright, J.G. Measurement of Mutual Diffusion Coefficients, Densities, Viscosities, and Osmotic Coefficients for the System KSCN-H_2O at 25 °C. *J Solution Chem* 1992, *21*, 1115-1129, doi:10.1007/BF00651858.
